# Using height-adjusted stunting prevalence will fail disadvantaged children worldwide

**DOI:** 10.1016/S2214-109X(22)00076-6

**Published:** 2022-04-12

**Authors:** Jef L Leroy, Edward A Frongillo, Elaine Borghi

**Affiliations:** a International Food Policy Research Institute, Washington, DC 20005, USA; b Department of Health Promotion, Education, and Behavior, University of South Carolina, Columbia, SC, USA; c WHO, Geneva, Switzerland

In their Comment (February, 2022),^1^ S V Subramanian and colleagues challenge the validity of using the prevalence of child stunting to monitor and evaluate nutrition policies. Their argument is based on a fundamental misunderstanding of the 2006 WHO growth standards and how to appropriately use and interpret stunting. The authors espouse a dangerous and unethical position.

The growth standards were constructed to show what we should expect if children grow in an environment favourable to growth, regardless of location. Consistent with this purpose, the Multicentre Growth Reference Study selected subpopulations living in conditions that do not constrain their growth.^2^ The study did not select children on the basis of maternal height as the authors suggest. Mothers being taller was the result of selecting children whose growth was not constrained, and it was not unique to India.

Maternal short stature is not simply passed down to children. The intergenerational association in height is driven by genetic and epigenetic mechanisms and the persistence of the deficient environment. Due to this persistence, adjusting for maternal stature underestimates the contribution of the current deficient conditions on a child's stature; therefore, it is flawed conceptually and statistically.

Furthermore, adjusting for maternal stature is unethical. Outcomes such as child development, educational attainment, and health status are also determined by parental (ie, genetic) factors and the environment that children grow in. Following Subramanian and colleagues’ logic, we would adjust for parental attainment or status for these outcomes as well. What the authors propose is equivalent to having lower expectations for children who have the misfortune of growing up in disadvantaged conditions. From the perspective of children's rights, all children in the world have the same right to grow and develop without constraints imposed by an inadequate environment. Having lower expectations for these children disregards the Sustainable Development Goals’ central commitment of leaving no-one behind and violates the ethical principles of beneficence (ie, the obligation to maximise benefits for children and to protect them from harm) and of distributive justice (ie, the need to treat children fairly and equitably).

Stunting has proven to be a remarkably useful metric for comparing the environmental deficits that children are exposed to between populations and over time.^3^, ^4^ Altering the way the prevalence of stunting is obtained and reported will create confusion. Adopting Subramanian and colleagues’ method will substantially disincentivise the commitment and actions that are urgently needed to improve the inadequate living conditions of hundreds of millions of children globally.

We declare no competing interests. The authors alone are responsible for the views expressed in this letter and they do not necessarily represent the views, decisions, or policies of the institutions with which they are affiliated.
